# Rheumatoid arthritis epidemiology: a nationwide study in Poland

**DOI:** 10.1007/s00296-024-05591-8

**Published:** 2024-04-27

**Authors:** Magdalena Krajewska-Włodarczyk, Mateusz Szeląg, Bogdan Batko, Zbigniew Żuber, Michał Orleański, Krzysztof Podwójcic, Jakub Sowiński, Jakub Jopek, Maria Świderek, Michał Maluchnik, Marek Brzosko, Agata Śmiglewska, Brygida Kwiatkowska

**Affiliations:** 1 Department of Rheumatology, School of Medicine, Collegium Medicum, University of Warmia and Mazury, Olsztyn, Poland; 2 Department of Analysis and Strategy, Ministry of Health, Warsaw, Poland; 3 Department of Rheumatology and Immunology, Faculty of Medicine and Health Sciences, Andrzej Frycz Modrzewski University, Kraków, Poland; 4 Department of Pediatrics, Faculty of Medicine and Health Sciences, Andrzej Frycz Modrzewski University, Kraków, Poland; 5 Institute of Labour and Social Studies, Warsaw, Poland; 6 Department of Adult Neurology, Medical University of Gdansk, Gdańsk, Poland; 7 Department of Rheumatology, Internal Diseases, Geriatrics and Clinical Immunology, Faculty of Medicine and Dentistry, Pomeranian Medical University, Szczecin, Poland; 8 Clinic of Early Arthritis, National Institute of Geriatrics, Rheumatology and Rehabilitation, Warsaw, Poland

**Keywords:** Rheumatoid arthritis, Epidemiology, Incidence, Prevalence

## Abstract

To assess the incidence and prevalence of rheumatoid arthritis (RA) in Poland for the period 2013–2021, total and dependent on gender, age, region and serological status. Information on reported National Health Fund (NHF) health services and reimbursed prescriptions were used, defining an RA patient as a person who had at least two visits in different quarters with ICD-10 code M05 or M06 and at the same time filled at least one reimbursed prescription for a drug whose active substance is methotrexate, sulfasalazine, leflunomide or was treated with biologic disease-modifying anti-rheumatic drugs (bDMRDs) or targeted synthetic DMARDs (tsDMARDs) as part of a drug program financed by the National Health Fund. The nationwide standardised incidence rate of RA in 2021 was 29 persons per 100,000 population (18 per 100,000 population of seropositive vs. 11 per 100,000 population of seronegative RA). The prevalence of RA in Poland in 2021 was 689.0 people per 100,000 population, a total of 0.7% (1.1% in women and 0.3% in men). The incidence of seronegative RA was approximately 38%. The majority of new RA diagnoses were in the sixth and seventh decades of life, irrespective of patients’ gender. The results allow RA to be classified as a disease with a significant social impact. A trend of later onset of RA has been observed, which requires special consideration of the needs of patients over 55 years of age.

## Introduction

Rheumatoid arthritis (RA) is a genetically and environmentally determined chronic disease characterised by numerous extra-articular complications in addition to progressive damage to the affected joints [1–3]. The systemic nature of RA results in a lower quality of life impaired social and occupational functioning and a significantly shorter life span compared with the general population [4]. Prompt diagnosis, based on the clinical manifestation, additional examinations and the experience of the rheumatologist, is essential to start appropriate treatment to achieve disease remission [5, 6]. Advancements in understanding the pathomechanisms responsible for the development of RA have allowed the creation of new treatment options that can improve prognosis. The majority of RA patients respond positively to conventional disease-modifying drugs, but some, particularly those with existing risk factors for poor prognosis, require innovative therapies. Difficult-to-treat rheumatoid arthritis (D2TRA) is still a huge problem, requiring specific monitoring of the disease activity and a specific therapeutic approach [7]. In 2016, WHO presented estimated data including years lived with disability (YLD), years of life lost (YLL) and disability-adjusted life years (DALYs) by age, sex and country [8]. The epidemiology of rheumatoid arthritis seems to be changing in recent decades. Epidemiological studies of global data on the incidence of RA are different depending on the estimation methods adopted, the size of the assessed groups and the country [8, 9]. Differences in RA incidence are observed between the assessed regions. Incidence has increased globally in recent decades. However, there has been a declining trend in disability-adjusted life years [10].

We aimed to estimate the incidence and prevalence of RA in Poland from 2013 to 2021 as accurately as possible, taking into account demographic factors, serological status, and possible regional differences.

## Materials and methods

We used electronic administrative health claims collected from 2009 to 2022 by the National Health Fund (NHF), a single public health care payer in Poland. The NHF database comprises individually reported data claimed to the payer by service providers. The data include detailed service descriptions and demographic variables describing the patient. The data collected in databases are anonymous, but individual patients can be distinguished and matched by their IDs, which are pseudonymized national identification numbers.

### Definition

Information on reported NHF health services and reimbursed prescriptions were used to identify people with seropositive rheumatoid arthritis (M05 with extensions) or other rheumatoid arthritis (M06 with extensions).

To eliminate initial diagnoses reported as M05 and M06 but not confirmed by further diagnosis, we defined patients with RA as individuals who had at least two visits in two different quarters (at least 90 days apart) with ICD-10 code M05 or M06 and filled at least one reimbursable prescription for a drug whose active substance is methotrexate (ATC L01BA01, ATC L04AX03), sulfasalazine (ATC A07EC01) or leflunomide (ATC L04AA13) or was treated with biologic disease-modifying anti-rheumatic drugs (bDMARDs) or targeted synthetic DMARDs as part of a drug program financed by the National Health Fund after being initially coded as RA.

The date of the second visit with code M05 or M06 was considered the date of the first diagnosis.

Registered incidence was defined as the number of new patients diagnosed with RA reported to the public health care system in a given calendar year. The registered prevalence in a given year was defined as the number of living patients on December 31 diagnosed earlier with RA in the health care system financed from public funds.

### Patients

Databases from 2009 to 2022 were used to identify patients and determine the date of first diagnosis. Due to access to reimbursement prescription data only from 2012, to avoid the error of overestimating the assessed RA incidence in 2012, we assessed incidence from 2013. The results were presented up to 2021, as one of the assumptions in the definition of a patient is the interval between subsequent visits with the ICD-10 code M05 or M06 of at least 90 days. In the case of 2022, some people were not able to meet the definition of a patient suffering from RA because they could make their next visit in 2023. For this reason, we decided that the incidence and prevalence recorded in 2022 as underestimated should not be presented in this work. However, data for 2022 were used in the analysis to estimate the incidence and prevalence in 2021 correctly.

The number of new RA diagnoses, divided into seropositive and seronegative RA, was also analyzed based on diagnosis codes M05 and M06 (with extensions). Due to the possibility of seroconversion, especially during the initial period of the disease, seropositive and seronegative cases were defined based on the diagnosis code of the last visit.

Patients’ place of residence was chosen based on the voivodship of their first visit with the ICD-10 code M05 or M06 (based on the TERYT code reported in their medical history). Only those with the correct TERYT code were included to determine the distribution of patients by voivodship.

### Statistics

All database querying, data processing, and computations, as well as visualisations, were carried out using the GNU R programming language within the RStudio environment. Discrete variables were summarized using counts and proportions (N, %).

The percentage of prevalence of RA in Poland was calculated on the basis of the presented NHF data and additional demographic data covering the number of Polish inhabitants made available by the Central Statistical Office [11].

To calculate standardized incidence and prevalence, we use data from the Central Statistical Office on the population size in a given year. We used standardized indicators for the entire Polish population to compare the incidence and prevalence in Poland with those of other highly developed countries.

## Results

### Incidence of RA

In 2021, 11,195 people were diagnosed with RA in Poland. The standardised incidence in Poland was 29 people per 100,000 inhabitants. In 2018, before the onset of the COVID-19 pandemic 2019, 40 people per 100,000 inhabitants were diagnosed with the disease. A reduction in the number of new cases since 2013 (52 per 100,000 inhabitants) was also observed in 2018—suggesting a decreasing trend in incidence; however, the interpretation of trends in incidence is difficult due to the limited time range (2009–2022) of the analysis.

Between 2013 and 2021, there was no change in the proportional distribution of both seropositive and seronegative forms of RA during this period. In 2013 and 2021, the incidence of seronegative RA was approximately 38% (Table 1).

**Table 1:** Registered incidence for seropositive and seronegative RA from 2013 to 2021

Year	Number of M05 cases	M05 per 100,000 person-years	Number of M06 cases	M06 per 100,000 person-years
2021	7059	18.5	4136	10.9
2020	6592	17.2	3990	10.4
2019	9006	23.5	5502	14.3
2018	9600	25.0	5790	15.1
2017	10,024	26.1	6116	15.9
2016	10,822	28.2	6424	16.7
2015	11,492	29.9	6928	18.0
2014	12,290	31.9	7514	19.5
2013	12,680	32.9	7724	20.1

**Fig. 1:**
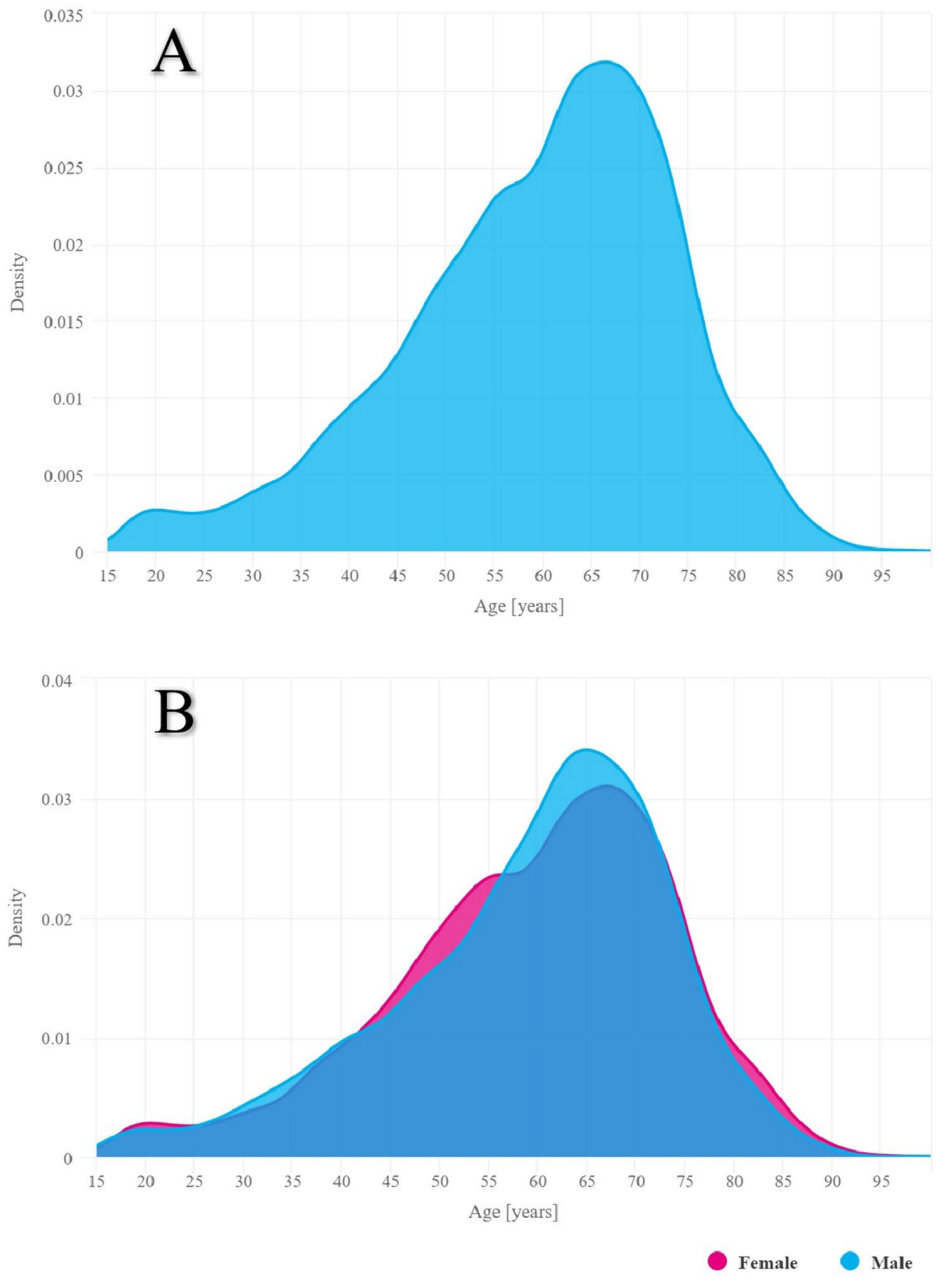
Age distribution of RA incidence. **A** Age distribution of RA incidence in the general Polish population in 2021. **B** Age distribution of RA incidence versus gender in the Polish population in 2021

The majority of new RA diagnoses in 2021 were in the sixth and seventh decades of life, irrespective of patients’ gender (Fig. 1). The mean age at which most new RA diagnoses were made in 2021 was 59.4 years, and the median age was 62 years (Table 2).

**Table 2:** Age distribution of patients diagnosed with RA for the first time between 2013 and 2021

Year	Number of patients	Percentage of women	Mean age (SD)	Median age (IQR)	Age range
2021	11,195	72	59 (14)	62 (50–70)	16–97
2020	10,582	72	59 (14)	61 (51–69)	16–100
2019	14,508	72	59 (14)	61 (51–69)	16–97
2018	15,390	73	59 (14)	61 (51–69)	16–97
2017	16,140	73	58 (14)	60 (50–68)	16–97
2016	17,246	74	58 (14)	60 (50–67)	16–103
2015	18,420	74	58 (14)	59 (50–67)	16–99
2014	19,804	75	57 (14)	59 (50–67)	16–95
2013	20,404	75	57 (14)	58 (50–66)	16–95

IQR, interquartile range; SD, standard deviation

In 2021, people living in urban and rural areas were most frequently diagnosed with RA at ages 67 and 61 years, respectively.

### The prevalence of RA in Poland

In 2021, 262,265 people in Poland were affected by RA. The standardized prevalence (per 100,000 inhabitants) across Poland was 689 people per 100,000 inhabitants. Among all voivodships, the standardized prevalence of RA ranged from 536 to 859 per 100,000 inhabitants.

The percentage of prevalence of RA in Poland in 2021 was 0.7%. The proportion of people of working age (18–65 years) diagnosed with RA accounts for 48% of the total number of patients.

**Fig. 2:**
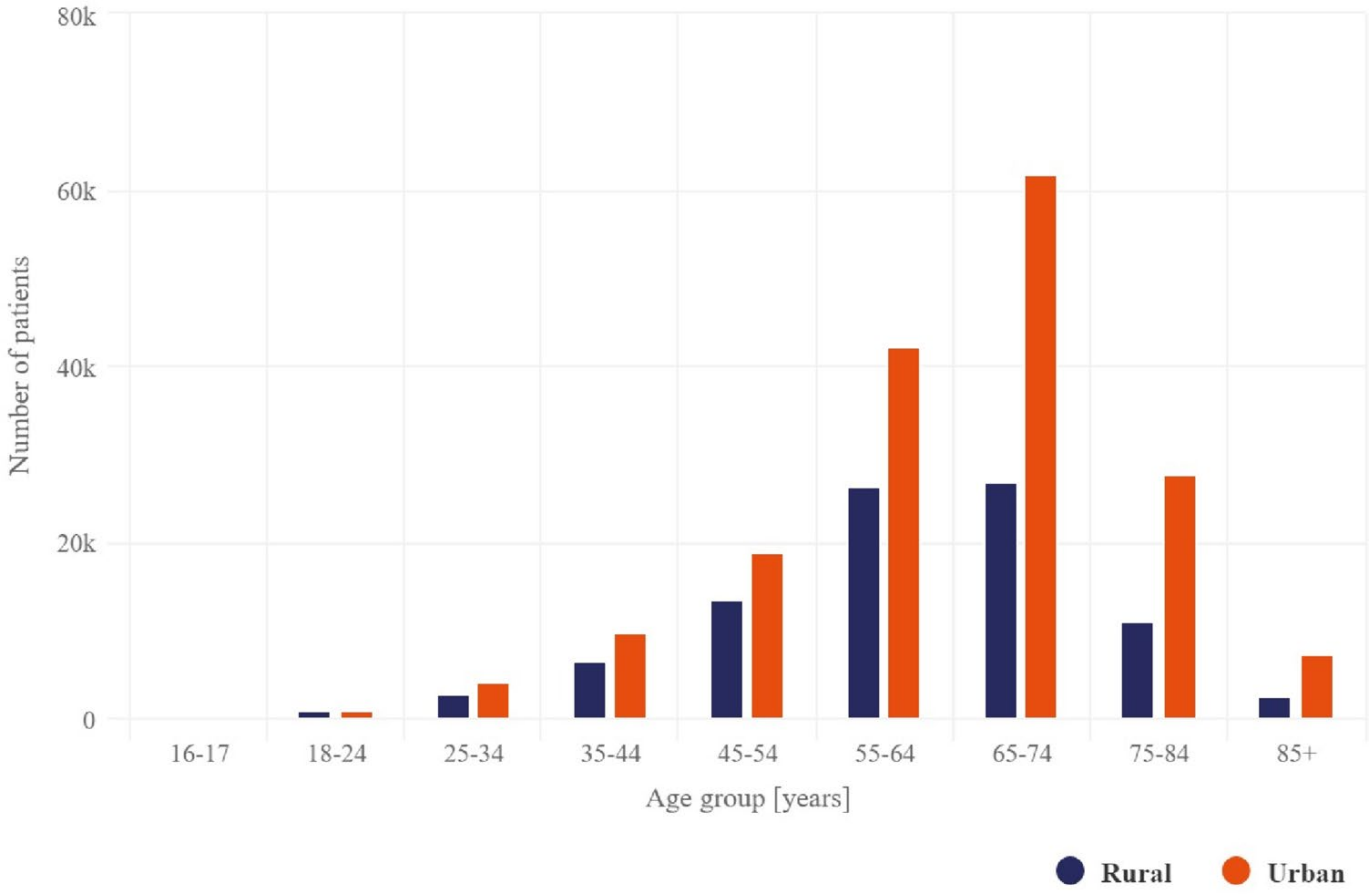
Number of all patients in Poland by age group and area of residence

There was no significant difference in the general prevalence of urban and rural residents. Most RA patients in Poland (approximately 66%) are urban residents (Fig. 2). This distribution is similar to Poland’s population distribution. However, the average age of patients living in urban areas is almost 3 years higher than those living in rural areas; additionally, the proportion of RA patients over 65 years of age living in urban areas is 57% and 45% in rural areas.

### Disease incidence and prevalence versus gender

**Fig. 3:**
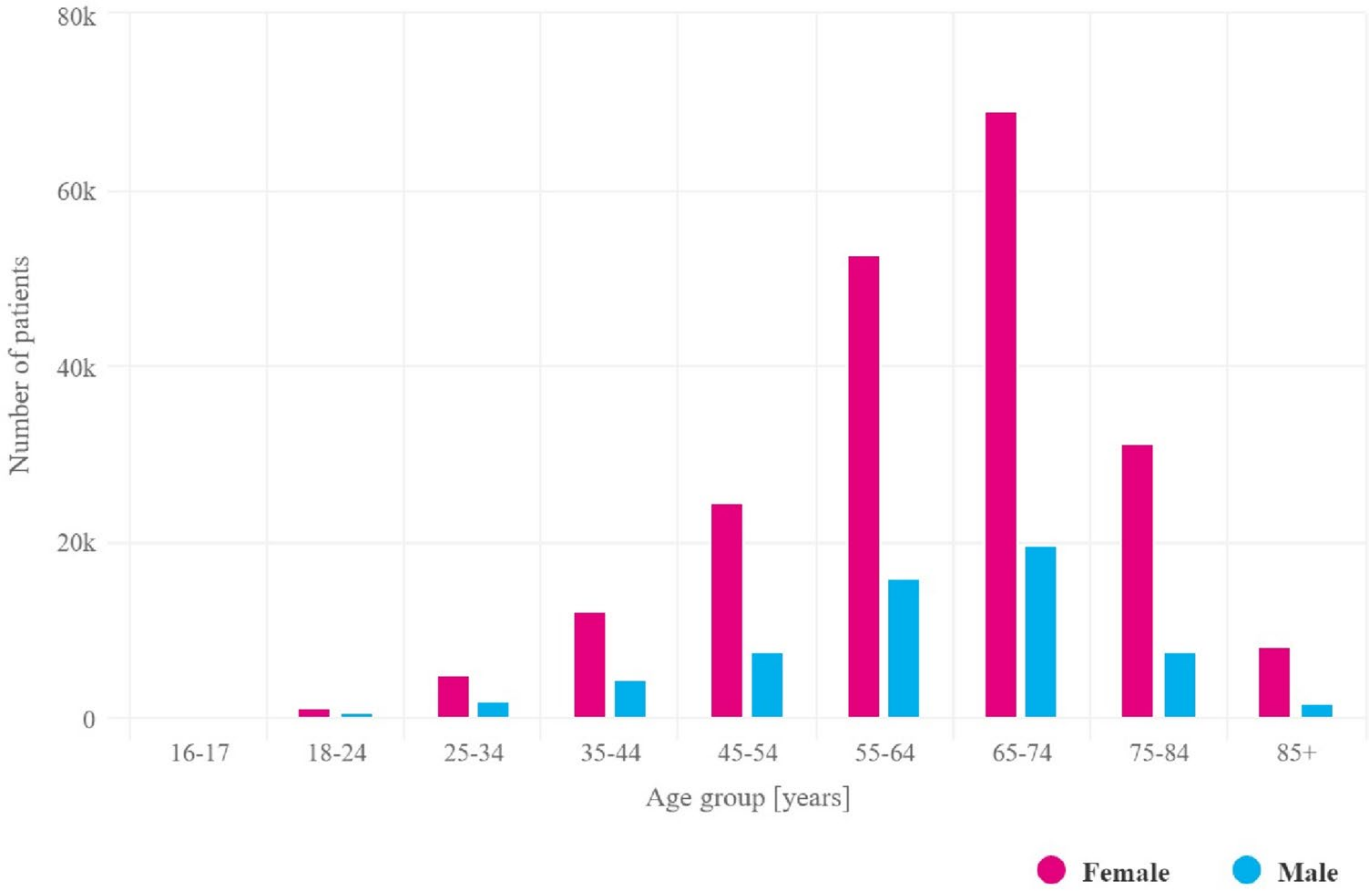
Number of all RA patients in Poland in 2021 by age group and gender

The peak incidence of RA in both men and women occurred in 2021 in the sixth and seventh decades of life (Fig. 1). No significant gender-related differences in the age of onset were observed throughout the study period; however, the disease was far more common in women (Fig. 3). The mean age at which most new RA diagnoses were made in 2021 was 59.5 years for women and 59.3 years for men.

In 2021, approximately 72% of the total number of people diagnosed with RA were women, with the percentage distribution changing with age. In the youngest patient group, up to 24 years of age, women accounted for almost 71% of patients, and after 85 years of age, over 83% (Table 3). The prevalence of the disease among women in 2021 was 1.1%, and among men, 0.3%.

**Table 3:** Age distribution of patients diagnosed with RA for the first time in 2021

Age range	Number of patients	Percentage of women	Median age (IQR)
18–24	217	71	21 (19–22)
25–34	408	66	31 (28–33)
35–44	998	71	40 (38–42)
45–54	1958	75	50 (48–52)
55–64	2995	69	60 (57–62)
65–74	3321	70	69 (67–72)
75–84	1132	73	78 (76–81)
85 +	140	83	87 (85–88)

IQR, interquartile range

## Discussion

The epidemiology of RA appears to be changing in recent years. Estimating such a significant health problem as RA requires obtaining information that is as close to real life as possible. Only complete knowledge allows the formulation of needs, management strategies and the personal and social costs of the disease. The development of proper outcomes, based on data available from patient registries, requires an adequate definition of patients classified as having or suffering from RA. The study presented here analyzes administrative data, including information on reported health services provided by the National Health Fund and reimbursed prescriptions filled between 2013 and 2021. It is the most extensive study assessing the prevalence of RA in Poland and, to our knowledge, one of the largest in Europe. It is also the first study evaluating the incidence of RA in the Polish population. The definition of an RA patient used for the study, to a large extent, allows for the elimination of initial and unconfirmed diagnoses reported as disease entities with ICD10 codes M05 and M06. Rheumatoid arthritis is a partly genetic, partly environmental disease. The elimination of certain modifiable environmental risk factors, such as cigarette smoking [12] or improved oral hygiene [13], may influence the reduction of incidence or delayed onset. It should be mentioned that the EULAR/ACR classification criteria proposed in 2010 simplified the diagnosis of early-onset RA compared with the earlier 1987 ACR criteria [14]. Based on the assumption that most cases of RA should be diagnosed within a few months of the first symptoms, the described peak incidence among people between 55 and 65 years of age may be influenced by a real-time delay in incidence related to the change in environmental factors mentioned earlier. However, it may also be a problem associated with a prolonged lack of established diagnosis in previously affected individuals as a result of prolonged symptomatic treatment prescribed in primary healthcare centres. This situation, caused on one hand by lack of referral and on the other by difficult access to a rheumatologist, especially in rural areas and small towns, clearly results in a significant delay in diagnosis, implementation of treatment and deterioration of the patient's general health. The prevalence of RA in the general Polish population was estimated at 0.7% (1.1% in women and 0.3% in men). The data obtained are consistent with previous results of a direct epidemiological study conducted in 2019 on a representative nationwide sample of 3000 Polish people, in which the prevalence was 0.9% [15]. The lower prevalence of RA presented in our study compared to the previously conducted Polish study may be related to a different methodology (using administrative data with a strict patient definition in our study). In this context, our results represent the lower limit of the actual prevalence, and it cannot be ruled out that there are slightly more RA cases in the Polish population. There are few current European studies on the prevalence of RA. Results similar to ours were reported in a Spanish study, where the estimated prevalence of RA was 0.82% [16] and in a recent study from the United Kingdom, where the prevalence of RA was estimated at 0.78% [17]. In a German study including four different case definitions, four different prevalence values standardised to the German population were obtained, ranging from 1.38 to 0.55% [18].

Data collection methods have a very strong influence on the results of the published analyses achieved. One of the highest prevalence rates in the world, 1.9% in the general Australian population, was provided by the Australian Bureau of Statistics (ABS) based on the 2017–2018 National Health Survey (NHS) (ABS 2018) [19], while much lower prevalence values of 0.8% (95% CI 0.8–1) were obtained using data from the NPS MedicineWise MedicineInsight dataset between 2000 and 2016 [20]. In contrast, using mathematical modelling, estimating missing data from nearby Oceania, the prevalence of RA was calculated to be 0.13% [10].

European population-based studies indicate a decline in the incidence of RA in recent years. A recent study by Scott et al. presented the results of RA incidence and prevalence in the UK population [17]. The authors used administrative data from the General Practice Research Database (GPRD) and, based on an algorithm that classifies a patient (reporting in services 2 × disease code or 1 × code plus prescribing a modifying drug) as suffering from RA, described a continued relatively constant incidence between 2013 and 2019, ranging from 49.1 (95% CI 47.7–50.6) to 52.1 (95% CI 50.6–53.6). The highest incidence was observed in those aged 65–75 years, and an increase in prevalence of more than 40% between 2004 and 2020 to 0.78% (95% CI 0.774–0.784). An earlier UK population study assessing patients with specific disease codes on the GPRD register revealed an annual decrease in incidence of 1.6% (95% CI 0.8–2.5) between 1990 and 2014 and an increase in prevalence of 3.7% per year (95% CI 3.2–4.1) between 1990 and 2005, with a subsequent decrease of 1.1% per year between 2005 and 2014 [21]. In our study, a reduction in the number of new cases from 2013 (52 per 100,000 inhabitants) to 2021 (29 per 100,000 inhabitants) was also observed—suggesting a decreasing trend in incidence. However, the interpretation of trends in incidence is difficult due to the limited time range (2009–2022) of the analysis and the period of the COVID-19 pandemic, which may have affected the reporting of administrative data. The number of new RA diagnoses varied between urban and rural areas.

Changes in epidemiology also seem to affect the incidence of seropositive and seronegative RA. Similar to our results, other studies have reported an equalisation of the incidence of seropositive and seronegative forms of RA [22, 23]. In a retrospective study by Myassoedova et al. including 427 patients diagnosed with RA between 2005 and 2014, rheumatoid factor (RF) was found in 51% and cyclic citrullinated peptide antibody (anti-CCP) in 50%. At the same time, they observed that in comparison with 1995–2004, a significant decrease in the proportion of seropositive RA cases in the whole group (from 69% for RF-positive RA cases), with an increase in the incidence of RF(−) RA (from 31% for RF-positive RA cases) occurred [22]. A considerable decline in the incidence of seropositive RA in the < 55 yrs group was found in a Finnish retrospective study covering 40 years. In 2020, the incidence of RF(+) RA in this study was 22.3 (95% CI 16.3 to 29.8)/100,000 inhabitants. There, no gender differences in the incidence of seropositive RA were found [23]. In contrast, another Finnish study, using data from the Social Insurance Institution of Finland's nationwide registry between 2000 and 2014, observed a decrease in the incidence of seronegative RA while the incidence of seropositive RA was maintained [24].

In our study since 2013, a trend of later onset of RA has been observed, with a slightly increasing but generally stable mean and median age at first diagnosis. The age of onset for all patients differed between urban and rural areas. Middle-aged and older women are a group that requires special attention. In the present analysis, women over 55 years accounted for as many as 60% of all RA patients, which certainly indicates the unique health needs of this patient group, including not only the treatment of the main disorder but also the treatment of concomitant diseases and the more common complications of the RA treatment used. Similar to our analysis, other recently published European studies have observed a later onset of RA, with the incidence in females about twice that in males in each age group and with a peak between 65 and 75 years [17] and even later, between 70 and 79 years [25]. Compared with previous reports involving a long-term follow-up period, the incidence of RA could differ by ten or even more years [23].

Studies published until now assessing RA worldwide vary according to sample size, data collection methods adopted and geographical location. Local healthcare systems, with common access to specialized care, play a significant role in assessing the prevalence of RA. The discrepancies in incidence and prevalence obtained in global studies can be a result of underestimation in certain regions, genetic predisposition and environmental factors [8]. The globally estimated incidence is lower than that estimated in European studies [8, 17, 21, 25]. Although global incidence data vary, attempts have been made to predict global trends in RA incidence [26]. The Bayesian Age-Period-Cohort Analysis (BAPC) results indicate an increase in the incidence of RA worldwide over the next few years at a moderate rate. According to the prognosis, by 2030, the global incidence of RA among women will increase to 18.23 per 100,000 people and among men to approximately 8.34 per 100,000 people. Despite regional differences, this prognosis, combined with the expected longer survival of patients, indicates a further increase in the global incidence of RA [26].

An advantage of the presented analysis is the development of results based on data on all RA patients diagnosed and treated in public health care in Poland between 2013 and 2021 and an approach that eliminates potentially unconfirmed disease diagnoses based on a strict case definition. To prepare an incidence study as reliable as possible, we checked the incidence of ICD 10 codes M05 and M06 since 2009 and estimated the incidence in 2021 only after obtaining complete data for 2022.

Our study has some limitations. The current analysis includes patients treated in public medical services only. It is possible that some patients were diagnosed before 2009; however, between 2009 and 2022 they did not use public specialist services, remaining exclusively under primary care, and it was not possible to identify them administratively as RA patients. According to our definition including specialised outpatient visits or hospitalisations in public health care, it is possible that, due to the current lack of administrative possibilities to identify diagnoses in private care, patients using only private care and filling reimbursed prescriptions after 2009 were also missed. However, it appears that the proportion of RA patients in Poland treated exclusively in primary or private care is marginal. The interpretation of trends in incidence and prevalence may be affected by the limited time frame of the analysis (2009–2022). The results are presented from 2013 to 2021, as we considered that this period would be necessary to meet our definition of a patient. The study design focused on the incidence and prevalence of RA and did not include aspects such as genetic, environmental, and socioeconomic risk factors for the development of RA. Aspects related to multimorbidity and comorbidity in RA were also not considered.

Data on the current prevalence and incidence of RA are extremely important for planning strategies to diagnose and manage the effects of this severe disease rapidly. Adequate case definition of the disease, based on more than a single diagnosis and analysis of the full data reported to the National Health Fund, allowed us to estimate the problem as reliably as possible. The high prevalence of RA, affecting around 1% of the general Polish population, allows RA to be classified as a disease with a significant social impact. Therefore, it is necessary to establish comprehensive management, including early diagnosis, prevention of disability, development of concomitant diseases and premature mortality, and to develop effective treatment strategies based on current recommendations, with particular emphasis on the needs of patients over 55 years of age.

## Data Availability

The data in this study were obtained with the permission of the Ministry of Health of the Republic of Poland from electronic databases of the National Health Fund. Datasets are not publicly available because they contain sensitive data at an individual level. Aggregated data may be requested from the Department of Analyses and Strategies in the Ministry of Health by the provisions on access to public information (a justification for the public interest is required).
